# Supplementary material for: Tead1a Initiates Transcriptional Priming Through the TEAD1a/YAP‐Notch1‐Spi1/Cebpα Axis to Promote Neutrophil Fate
Source: Adv Sci (Weinh). 2025 Aug 28;12(41):e05441. doi: 10.1002/advs.202505441 (PMC12591127; doi:10.1002/advs.202505441)
Supplement: Supplementary file 4 — Supplementary Dataset [file ADVS-12-e05441-s004.zip › 3-7.zip!/3-7/02-7.cluster/cluster19/Enrich/ego_MF.pdf]

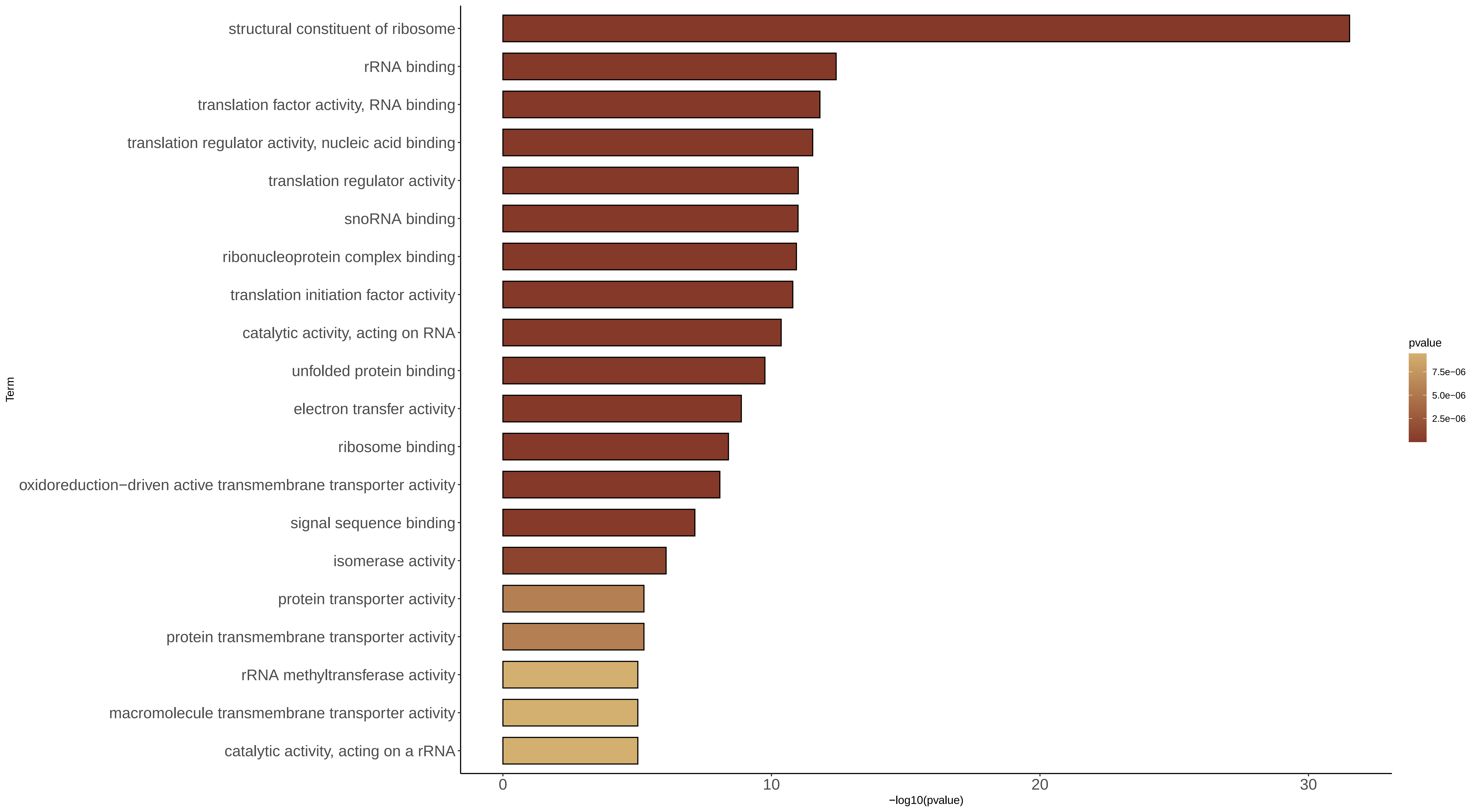
structural constituent of ribosome
rRNA binding
translation factor activity, RNA binding
translation regulator activity, nucleic acid binding
translation regulator activity
snoRNA binding
ribonucleoprotein complex binding
translation initiation factor activity
catalytic activity, acting on RNA
unfolded protein binding
electron transfer activity
ribosome binding
oxidoreduction−driven active transmembrane transporter activity
signal sequence binding
isomerase activity
protein transporter activity
protein transmembrane transporter activity
rRNA methyltransferase activity
macromolecule transmembrane transporter activity
catalytic activity, acting on a rRNA
Term
0
10
20
30
−log10(pvalue)
pvalue
7.5e−06
5.0e−06
2.5e−06
